# Overexpression of *ATG5* Gene Makes Granulocyte-Like HL-60 Susceptible to Release Reactive Oxygen Species

**DOI:** 10.3390/ijms21155194

**Published:** 2020-07-22

**Authors:** Agnieszka Mroczek, Adrianna Cieloch, Aneta Manda-Handzlik, Weronika Kuźmicka, Angelika Muchowicz, Małgorzata Wachowska

**Affiliations:** 1 Department of Laboratory Medicine and Clinical Immunology of Developmental Age, Medical University of Warsaw, Zwirki i Wigury 63a Street, 02-091 Warsaw, Poland; askrobot@wum.edu.pl (A.M.); adrianna.cieloch@gmail.com (A.C.); aneta.manda-handzlik@wum.edu.pl (A.M.-H.); weronika.kuzmicka@wum.edu.pl (W.K.); 2 Postgraduate School of Molecular Medicine, Medical University of Warsaw, Zwirki i Wigury 61 Street, 02-091 Warsaw, Poland; 3 Department of Immunology, Medical University of Warsaw, Jana Nielubowicza 5 Street, 02-097 Warsaw, Poland; angelikamuchowicz@gmail.com

**Keywords:** ATG5, autophagy, neutrophils, neutrophils functions, promyelocytic leukemia cells (HL-60)

## Abstract

Neutrophils represent the first line of defense against pathogens using various strategies, such as phagocytosis, production of reactive oxygen species (ROS) and neutrophil extracellular traps (NETs) formation. Recently, an autophagy-independent role of autophagy related (*ATG*) gene 5 in immune cells, including neutrophils, was emphasized. Our aim was to investigate the role of ATG5 protein in neutrophils’ antimicrobial functions, proliferation and apoptosis. To this end, we used genetically modified human promyelocytic leukemia (HL-60) cells overexpressing ATG5, differentiated toward granulocyte-like cells with all-trans retinoic acid (ATRA) and dimethylformamide. The level of differentiation, phagocytosis, proliferation and apoptosis were determined by flow cytometry. ROS production and NETs release was assessed by fluorometry and fluorescent microscopy. *ATG5* gene expression was evaluated by real-time PCR, whereas the protein level of ATG5 and LC3-II was determined by Western blot. We did not observe the induction of autophagy in differentiated HL-60 cells overexpressing ATG5. The increased expression of ATG5 affects the differentiation of HL-60 cells with ATRA, ROS production and phagocytosis. However, we did not detect changes in NETs release. Moreover, ATG5 protects differentiated HL-60 cells from apoptosis but does not cause changes in proliferation rate.

## 1. Introduction

In the last two decades, scientists have begun to pay attention not only to adaptive but also to innate immunity as a key player in the outcome of various diseases. It is well documented that the crucial components of the innate arm of immune response are neutrophils, the most abundant type of white blood cells in human blood. Neutrophils are directed by cytokines and other stimuli into infected tissues, where they eliminate invading microbes [1]. The role of these cells was underestimated for a long time, though recent research uncovered that neutrophils are sophisticated immune cells. They are able to precisely control enzymes in the granules, release chemokines and interact with several constituents of the immune system, including the adaptive branch. Neutrophils eliminate microbes through a variety of mechanisms such as phagocytosis, the production of reactive oxygen species (ROS), degranulation and neutrophil extracellular trap formation (NETs) [2].

Interestingly, studies of the last decade have identified autophagy as one of vital processes involved in neutrophil functions and bone marrow differentiation [3]. However, there are also data pointing to the potent autophagy-independent roles of proteins involved in autophagy, such as autophagy-related (ATG) 5 protein in neutrophils [4,5]. ATG5 was initially described as a crucial element in a process of autophagy. This membrane-trafficking mechanism is responsible for the turnover of cytoplasmic constituents and also serve as a cell survival and cell death pathway [6,7]. The process of autophagy is mostly controlled by *ATG* genes. Among them, the *ATG5* is essential for the early stages of autophagosome formation and for many years it was suggested that ATG5 protein is specifically required for autophagy [8]. However, a growing body of evidence shows that ATG5, as well as other components of the autophagy machinery, may be crucial elements in other processes [9]. It was reported that ATG5 plays a critical role in multiple aspects of lymphocyte development and function. Murine CD4 and CD8 lymphocytes lacking ATG5 failed to undergo efficient proliferation after TCR stimulation [10]. Intriguingly, ATG5 can eliminate pathogens by regulating cytokine secretion through crosstalk with various pathways. Moreover, there is direct evidence that ATG5 is involved in apoptosis. Calpain-cleaved ATG5 is further translocated to the mitochondria and triggers cytochrome c release together with caspase activation, without inducing the autophagy process [11].

Neutrophils are short-lived cells and any genetic manipulations are impossible to introduce by currently available techniques. Therefore, in the present study we used a differentiated human promyelocytic leukemia (dHL-60) cell line, recently described and characterized by us, resembling neutrophils’ functions [12]. Here, we developed the HL-60 cell line overexpressing *ATG*5, differentiated with all-trans retinoic acid (ATRA) or dimethylformamide (DMF), in order to examine the ATG5 role in neutrophils’ functions.

## 2. Results

### 2.1. ATG5 Overexpression in HL-60 Cells Does Not Result in the Upregulation of Autophagy

**Figure 1:**
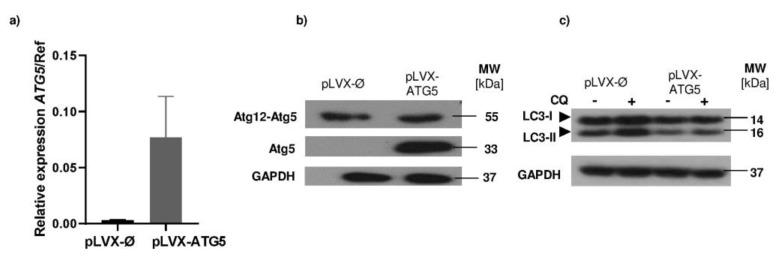
ATG5 overexpression in HL-60 cells does not result in the upregulation of autophagy. (**a**) HL-60 cells stably transduced with vector containing human ATG5 sequence (pLVX-IRES-Puro-ATG5) and control vector (pLVX-Ires-Puro-Ø, mock control) were collected and total RNA was isolated and reverse transcribed into cDNA. qPCR with LightCycler 480 SYBRGreen I Master kit was performed to determine the mRNA level of *ATG5* vs reference genes (β-actin (*ACTB*) and hypoxanthine–guanine phosphoribosyltransferase (*HPRT*)) in stably transduced HL-60 cell lines. (**b**) ATG5 overexpression in stable HL-60-ATG5 and mock control cell lines was confirmed by Western blotting. The band at 53 kD corresponds to the ATG12-ATG5 conjugate and the 33 kDa band corresponds to the unbound ATG5 form. (**c**) The HL-60 cells were incubated with 50 µM CQ (chloroquine) for 16 h and the level of accumulated LC3-II protein was evaluated by western blotting. MW—molecular weight, GAPDH—glyceraldehyde 3-phosphate dehydrogenase—served as a loading control. (**b**,**c**) Representative results are shown for one out of four independent experiments.

In order to investigate the role of autophagy/ATG5 in neutrophils functions, HL-60 cells were transduced with a lentiviral expression system. Vectors containing the human *ATG5* gene sequence—pLVX-IRES-Puro-*ATG*5 or control vector—pLVX-IRES-Puro-Ø were used. A stable cell line overexpressing ATG5 (HL-60-ATG5) and mock control cells, was obtained by a puromycin selection. The level of increased *ATG5* expression was measured at mRNA level by quantitative (q) PCR (Figure 1a) and at the protein level by Western blotting (Figure 1b). Our results confirm that the mRNA level of *ATG5* was increased in the HL-60-ATG5 cells, comparing with its mock control. Consequently, the protein level of ATG5 was strongly elevated in the HL-60-ATG5 cells, as we were able to detect the 33kDa monomer, which was not present in mock control cells. Subsequently, to determine the effect of increased ATG5 expression on the level of autophagy, mock control HL-60 cells and HL-60-ATG5 cells were incubated for 16 h with 50 μM chloroquine (CQ), an inhibitor of autophagosome–lysosome fusion. The conversion of LC3-I to LC3-II was assessed by Western blotting. In all cells incubated with CQ, we observed an increased conversion of the LC3-I protein to the LC3-II form when compared to untreated cells. However, we failed to observe stronger accumulation of LC3-II in HL-60 cells with increased ATG5 expression in comparison to mock HL-60 cells after incubation with CQ (Figure 1c) proving lack of autophagy upregulation in HL-60 cells overexpressing ATG5.

### 2.2. Increased Expression of ATG5 Affects Differentiation of HL-60 Cells with ATRA

**Figure 2:**
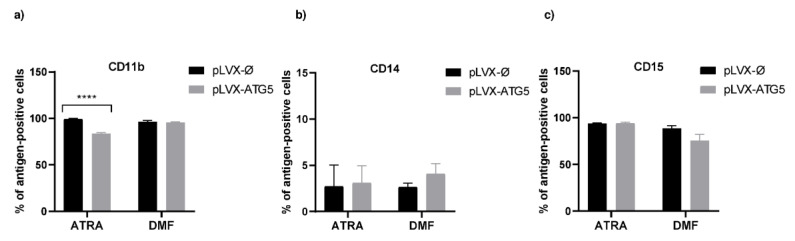
Increased expression of ATG5 affects the all-trans retinoic acid (ATRA)-induced differentiation of HL-60 cells. HL-60-ATG5 and mock control cells were differentiated into granulocyte-like cells by treatment with 1 µM ATRA or 70 mM dimethylformamide for 5 days. (**a**–**c**) Cells were harvested, stained with appropriate antibodies and differentiation was assessed by evaluating surface expression level of CD11b, CD14 and CD15 by flow cytometry. The data are presented as the means + SEM, *n* = 5 where *n* is the number of replicate experiments. **** *p* ≤ 0.0001 versus mock control, Kruskal–Wallis test with post-hoc Dunn’s test.

Mock control HL-60 and HL-60-ATG5 cells were cultured in the presence of ATRA or DMF for 5 days. Flow cytometry analysis revealed that both agents, ATRA and DMF, efficiently differentiated HL-60 cells toward granulocyte-like cells (% of CD11b cells: 94 ± 1.3% in DMF-dHL-60-ATG5 cells; 90 ± 1.4% in ATRA-dHL-60-ATG5 cells). However, mock control HL-60 cells differentiated with ATRA were characterized by significantly higher expression of CD11b than ATRA-dHL-60-ATG5 (Figure 2a). Importantly, we did not observe the presence of monocytic marker, CD14, on the surface of differentiated cell lines (Figure 2b), whereas CD15 expression was constant before and after differentiation (Figure 2c).

### 2.3. Increased Expression of ATG5 in Granulocyte-Like Cells Does Not Affect Ability to Form NETs

**Figure 3:**
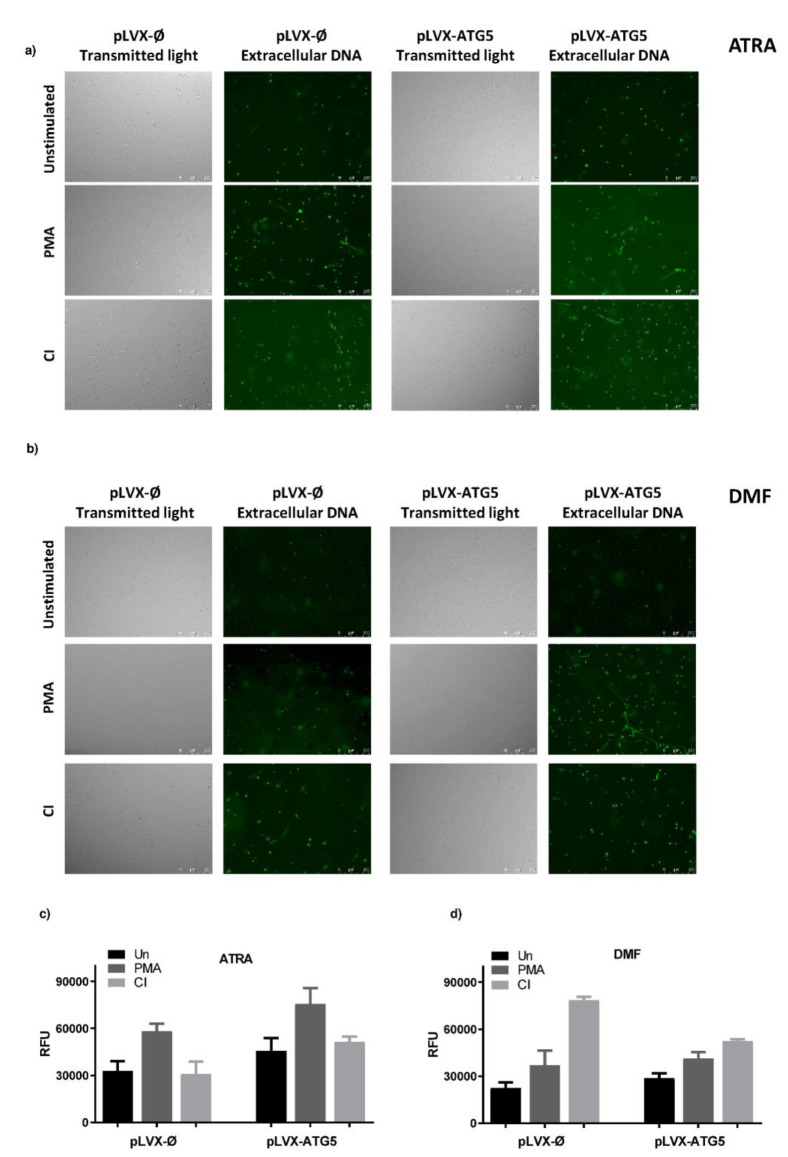
Increased expression of ATG5 in granulocyte-like cells does not affect their ability to form neutrophil extracellular traps (NETs). HL-60-ATG5 and mock control HL-60 cells were differentiated for 5 days with 1 µM ATRA and 70 mM DMF and stimulated with 100 nM phorbol 12-myristate 13-acetate (PMA) or 4 µM calcium ionophore A23187 (CI) or left unstimulated for 3 h. Neutrophil extracellular traps (NETs) formation was assessed qualitatively: (**a**,**b**) by conventional fluorescent microscopy and quantitatively (**c**,**d**) by fluorometric measurement of DNA release. (**a**,**b**) After the stimulation, 100 nM Sytox Green was added to the wells and NETs were visualized under the microscope using fluorescent and transient light, at 10× magnification. Representative images of one out of six independent experiments are shown. Results are shown as means + SEM from 6 independent experiments and were analyzed by one-way ANOVA with post hoc Dunn’s test.

To characterize neutrophils functions in dHL-60 cells overexpressing ATG5, we first determined their ability to release NETs. ATRA- and DMF-differentiated mock control and HL-60-ATG5 cells were stimulated for 3 h with phorbol 12-myristate 13-acetate (PMA) and calcium ionophore A23187 (CI). The measurement of extracellular DNA by fluorometry revealed that in ATRA-dHL-60 cells, PMA is a stronger NETs inducer than CI, however there was no statistically significant difference in the ability to form NETs between examined cell lines. In DMF-dHL-60 cells, the highest amount of DNA was released after CI stimulation and, similarly to ATRA-dHL-60, tested cell lines released comparable amounts of DNA (Figure 3c,d). In addition, the qualitative analysis of NETs release was carried out using fluorescent microscopy. This analysis of in vitro NETs release confirmed observations based on the quantitative method (Figure 3a,b).

### 2.4. ROS Production Is Augmented in ATRA-dHL-60-ATG5 Cells

**Figure 4:**
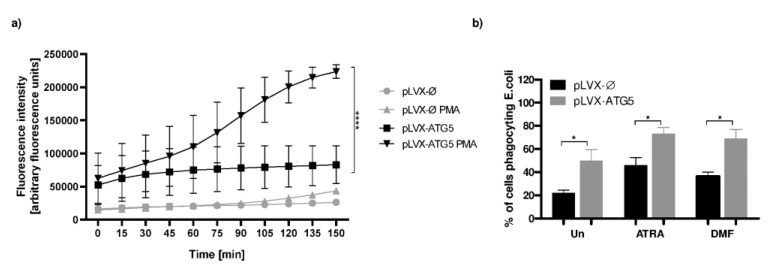
Increased expression of ATG5 leads to augmented reactive oxygen species (ROS) production and increased phagocytosis level in dHL-60-ATG5 cells. (**a**) The mock control HL-60 and HL-60-ATG5 cells after differentiation with 1 µM ATRA were loaded with dihydrorhodamine (DHR) 123 and left unstimulated or stimulated to release NETs with 100 nM PMA. Fluorescence was monitored every 15 min for 150 min post stimulation. (**b**) Mock control HL-60 and HL-60-ATG5 cells differentiated with 1 µM ATRA or 70 mM DMF were incubated with fluorescently labeled 25 µg *E. coli* bioparticles and after a 30-min incubation the percentage of phagocyting cells was measured with a flow cytometer (**a**,**b**) The means + SEM from 5 independent experiments or (**b**) 7 independent experiments are shown, (**a**) two-way ANOVA with Dunnett’s correction for multiple comparisons or (**b**) one-way ANOVA with post-hoc Newman-Keuls test. * *p* ≤ 0.05, **** *p* ≤ 0.0001

Next, as ATRA-dHL-60 cells reveal higher ability to generate ROS than HL-60 cells differentiated with polar planar compounds including DMF [12,13], we studied an oxidative burst in ATRA-dHL-60 cells overexpressing ATG5 and in mock control H-60 cells. To this end, we used dihydrorhodamine 123 (DHR) fluorescent probe, nonfluorescent ROS indicator which is oxidized to cationic rhodamine 123 exhibiting green fluorescence. Fluorometric analysis of oxidized DHR123 in ATRA dHL-60 cells lines indicated that ROS formation after PMA stimulation is significantly higher in cells overexpressing ATG5 than in mock control dHL-60 cells (Figure 4a).

### 2.5. Phagocytosis Level Tends to Be Elevated in dHL-60 Cells Overexpressing ATG5

Furthermore, we investigated whether overexpression of the *ATG5* gene in dHL-60 cells may affect phagocytosis, another antimicrobial function, besides NET formation and oxidative burst. Interestingly, we observed that undifferentiated HL-60 cells with increased expression of the *ATG5* gene reveal statistically significantly higher ability to phagocyte *E. coli* particles when compared to mock control. In addition, similar observations were made for ATRA-differentiated and DMF-differentiated HL-60 cells (Figure 4b).

### 2.6. Increased Expression of ATG5 Does Not Influence the Proliferative Potential of HL-60 Cells

**Figure 5:**
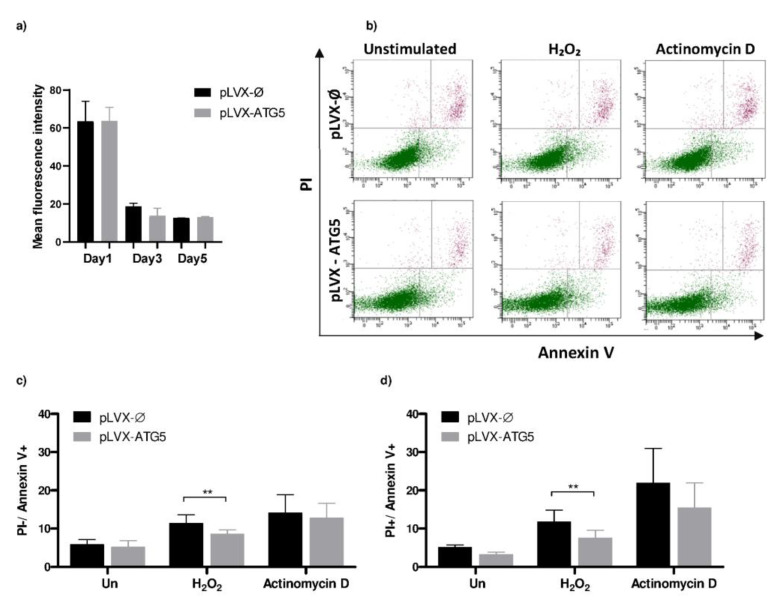
HL-60-ATG5 cells do not reveal altered proliferative potential but are more resistant to chemically induced apoptosis. (**a**) Mock control and HL-60-ATG5 cells were stained with 1 µM VPD450 dye and incubated for 5 days. Proliferation rate was assessed by flow cytometry analysis of median fluorescence intensity (MFI) on the first, third and the fifth day of the experiment. The data are representative of one out of five independent experiments. (**b**–**d**) Mock control and HL-60-ATG5 cells were incubated in the presence of 10 µM H_2_O_2_ or 1 µg/mL actinomycin D for 16 h, stained using fluorescein isothiocyanate (FITC)-conjugated annexin V and propidium iodide (PI) and analyzed by flow cytometry. (**b**) Representative dot plots. Percentage of early (**c**) and late (**d**) apoptotic cells. The data are presented as means + SEM of five independent experiments. ** *p* ≤ 0.01, two-way ANOVA with post-hoc Holm–Šídák’s test.

The proliferative potential of transduced cell lines was compared using Violet Proliferation Dye (VPD450) and flow cytometry. Analysis was carried out on the 1st, 3rd and 5th day of the experiment. The proliferation rate of tested cells lines on the 3rd and 5th day of analysis was comparable, as the mean fluorescence values were similar for both HL-60-ATG5 and mock control HL-60 cells (Figure 5a). These results suggest that the increased expression of ATG5 does not affect the proliferation of HL-60 cells.

### 2.7. HL-60-ATG5 Are More Resistant to Chemical Apoptosis Inducers

It was highlighted that ATG5 protein is also engaged in the regulation of apoptosis [9] and may protect from H_2_O_2_-induced apoptosis [14]. Therefore, we incubated HL-60 cells with two distinct commonly used apoptosis inducers (actinomycin or H_2_O_2_). Subsequently, phosphatidylserine (PS) plasma membrane externalization, one of early stages of apoptosis, was evaluated by annexin V/propidium iodide (PI) and analyzed by flow cytometry. Interestingly, the percentage of early and late apoptotic cells was lower in cells overexpressing ATG5 than in mock control in both actinomycin D-treated and H_2_O_2_-treated groups, whereas statistical significance was observed only for H_2_O_2_ (Figure 5b,c). Our results suggest that ATG5 may play a protective role against cell death in HL-60 cell line.

## 3. Discussion

It is well documented that neutrophils play an invaluable role in immune host defense. Inherited or acquired neutropenia results in severe infections, underlining the key role of neutrophils in fighting the pathogens. Even though their role in the immunity is invaluable, we still know relatively little about their function when compared to other immune cells [15].

The aim of this study was to evaluate the role of ATG5 in the antimicrobial functions of granulocyte-like cells such as NETs formation, ROS production and phagocytosis. To that end, we employed a model based on a genetic modification of granulocytic-like cells. We developed the HL-60 cell line overexpressing ATG5 and differentiated it with ATRA or DMF into granulocytic-like cells. Even though ATG5 level was increased both at the mRNA and at the protein level, we found that overexpression of ATG5 in HL-60 cells does not lead to induction of autophagy. Importantly, we observed significant differences in ROS production and phagocytosis level following the overexpression of ATG5, but not in the ability to release NETs, between dHL-60 cells overexpressing ATG5 and mock control cells. Moreover, we did not detect differences in the proliferation rate of HL-60-ATG5 when compared to mock control. However, we noticed that HL-60-ATG5 are more resistant to apoptosis inducers.

Autophagy is based on the formation of distinctive double-membrane vesicles, termed autophagosomes, and further lipidation of the LC3-I protein to its membrane-bound LC3-II form [16]. As it was mentioned before, autophagy-related proteins take part in this process, among which ATG5 is indispensable for autophagic vesicles formation [11]. It was reported that the overexpression of ATG5 in different cell lines resulted in the presence of 33kDa ATG5 monomer and led to the induction of autophagy, as measured by the lipidated form of LC3, i.e., LC3-II [17]. Similarly, in our studies the overexpression of ATG5 in HL-60 cell line was detectable, since we observed increased ATG5 both at mRNA and protein level. Western blot analysis clearly shows an additional 33 kDa band for ATG5 monomer in HL-60-ATG5 cells. However, in contrast to previous studies [18,19] we were not able to detect enhanced accumulation of LC3 II in cells overexpressing ATG5. Such discrepancies may arise from cell line model used in our experimental settings, as the HL-60 cell line was suggested to be defective in the ability to induce autophagy [20].

It was demonstrated several times that the ATRA-induced granulocyte differentiation of acute promyelocytic leukemia cell line NB4 and/or HL-60 cells upregulates autophagy. The pharmacologic inhibition of autophagy attenuates the ATRA-induced differentiation of NB4 and/or HL-60 cells [21,22]. Moreover, it was suggested that the expression of autophagy-related genes such as *BECN1*, *ATG1* or *ATG5* is upregulated after ATRA treatment in NB4 and/or HL-60 cells [23]. Therefore, it was interesting to evaluate whether increased availability of the ATG5 protein may influence the ability of ATRA and DMF to differentiate HL-60 into granulocytic-like cells. Flow cytometry analysis of surface marker CD11b revealed that both HL-60-ATG5 and mock control HL-60 cells can be effectively differentiated, by ATRA and DMF, into granulocyte-like cells. Unexpectedly, dHL-60-ATG5 cells revealed lower expression of CD11b than mock control only after ATRA treatment. Since ATG5 expression may be increased in ATRA differentiated cells [23], we assume that the level of ATG5 protein in ATRA dHL-60-ATG5 cells is higher than in DMF-dHL-60-ATG5 cells. Therefore, we can speculate that increased ATG5 accumulation in ATRA-treated HL-60 cells may lead to the excessive protein degradation in these cells, but not in DMF-treated HL-60.

Interestingly, it was suggested that ATG5 itself may activate neutrophils as well as interact with MyD88 [11]. MyD88 is a signalling adaptor molecule and an important player in activating nuclear factor (NF)-κB signalling and mitogen-activated protein kinase signalling cascades. Furthermore, it is responsible for the transcription of many genes associated with innate immunity [11]. Therefore, we hypothesized that ATG5 overexpression may influence the antimicrobial function of granulocytes. To verify this hypothesis, we evaluated neutrophils functions, such as phagocytosis, and ROS production as well as NETs formation, in ATRA- and DMF-differentiated HL-60-ATG5 cells.

There are contrary data concerning ATG5 role in NETs formation. It was reported that downregulation of ATG5 impairs NETs formation [24] whereas others showed that murine neutrophils lacking the *Atg5* gene and ATG5-dependent autophagy are able to form NETs [25]. In our study, we were unable to detect differences in the ability to release NETs between dHL-60 overexpressing ATG5 and mock control cell line. There are data suggesting the relationship of ATG5 with ROS production, for example, Tal et al. showed that ATG5−/− cells accumulated ROS localized to the mitochondria [26], whereas Pyo et al. described that transgenic mice overexpressing Atg5 revealed higher levels of antioxidants than their control littermates [27]. In our study, the increased expression of ATG5 in ATRA dHL-60 led to increased basal level of ROS and further enhanced ROS production after PMA stimulation. These observations suggest that the aberration of ATG5 level influences ROS production. Moreover, it was demonstrated that silencing of *ATG5* by siRNA leads to a reduction in the neutrophils phagocytosis rate [28]. Our results are in line with this report, as we observed increased phagocytosis of *E. coli* particles by differentiated (ATRA and DMF) HL-60 overexpressing ATG5 in comparison to mock control.

It was recently reported that the role of ATG5 is much broader than initially thought, as it occurred that, besides playing a vital role in autophagy, ATG5 also affects the cell proliferation rate and apoptosis. In studies done by Zheng et al., the increased expression of ATG5, achieved by viral transduction led to the increased proliferation of tested cell lines [19]. Our results revealed that the enhanced expression of ATG5 did not affect the proliferation rate of HL-60 cells. This feature may be cell-line-dependent. Interestingly, it was described by Weng et al. that the overexpression of ATG5 protects cells from H_2_O_2_-induced apoptosis [14]. Our results are in line with already existing data. In comparison to mock control, HL-60-ATG5 cells were less susceptible to PS membrane externalization, an early stage apoptosis marker, after treatment with H_2_O_2_.

Altogether, here we report that the induction of autophagy by the overexpression of *ATG* genes may not be achieved with all types of cell lines, since we did not observe such a phenomenon in HL-60 cell line, while previous reports showed such dependence. ATG5 was previously described as an immunomodulatory agent, independently from its autophagy-related role. Our data are in line with these observations, as we showed that an excess of ATG5 protein facilitates ROS production and influence phagocytosis. Moreover, an excess of ATG5 itself may protect HL-60 cells from apoptosis.

## 4. Materials and Methods

### 4.1. Reagents

Fetal bovine serum (FBS) was purchased from Biochrom (Berlin, Germany). Roswell Park Memorial Institute (RPMI) 1640 medium, HEPES, SYTOX Green, SYTOX Orange, *E. coli* (K-12 strain) BioParticles, dihydrorhodamine (DHR) 123 were obtained from Thermo Fisher Scientific (Waltham, MA, USA). Anti-CD11b- phycoerythrin (PE) (IM2581U) was from Beckmann Coulter (Brea, CA, USA). Anti-CD15-PE-Cy7 (560827) antibody and VPD 450 (562158) were purchased from BD Bioscience (San Jose, CA, USA) and anti-CD14-alexa fluor (AF) 750 (FAB3832S) from R&D System (Minneapolis, MN, Canada). Anti-neutrophil elastase (NE) (ab21595), anti-myloperoxidase (MPO) (ab11729) and secondary anti-rabbit horseradish peroxidase (HRP)-conjugated (ab97051) antibodies were obtained from Abcam (Cambridge, UK). Anti-LC3A/B (#4108), anti-ATG5 (#129945), secondary anti-rabbit (#7074) conjugated with HRP antibodies were purchased from Cell Signaling Technology (Beverly, MA, USA). Anti-glyceraldehyde 3-phosphate dehydrogenase (GAPDH) antibody (G9295), anti-β actin (ACTB) antibody (A3854), HL-60 cells (98070106), bovine serum albumin (BSA), calcium ionophore A23187 and all other reagents, unless otherwise stated, were purchased from Sigma Aldrich (St Louis, MO, USA).

### 4.2. Cell Culture and Differentiation

Human embryonic kidney epithelial cells (HEK293T) and human promyelocytic leukemia cells HL-60 were cultured in Roswell Park Memorial Institute (RPMI) 1640 medium supplemented with 10% FBS and antibiotic-antimycotic solution at 37 °C, 5% CO_2_. HL-60 cells were differentiated with 1 µM ATRA or 70 mM DMF, as described previously [12]. Level of HL-60 cells differentiation was evaluated by flow cytometry analysis of CD11b, CD14 and CD15 expression using a BD LSRFortessa Flow Cytometer and BD FACSDiva Software. The viability of differentiated cells was determined using a trypan blue exclusion assay. For all further studies, cells were suspended in protein-free RPMI 1640 medium without phenol red supplemented with 10 mM HEPES.

### 4.3. Plasmid Construction

*ATG5* sequence was multiplied by PCR using commercially available plasmid pEGFP-C1-hATG5 with the forward and reverse primers containing restriction sites for Not I and Bam H I, respectively (Table 1). The PCR product was digested and cloned into the multicloning site of the puromycin-resistant mammalian expression vector pLVX-IRES-Puro. The sequence of the pLVX-IRES-puro-ATG5- construct was confirmed by DNA sequencing.

### 4.4. Generation of HL-60 Cells Line Stably Overexpressing ATG5 Gene

HL-60 cells stably overexpressing human *ATG*5 were generated using a second-generation lentiviral system. HEK 293T cells were co-transfected with pLVX-IRES-Puro-ATG5 or pLVX-IRES-Puro, packaging psPAX2 and envelope pMD2.G vectors using GeneJuice transfection reagent (Calbiochem, San Diego, CA, USA), according to the manufacturer’s instruction. Forty-eight hours post-transfection, lentiviruses-containing medium from the HEK 293T cells was collected, filtered and HL-60 cells were infected in the presence of 8 µg/mL Polybrene. Stable cell line was obtained by puromycin (10 µg/mL) selection, and overexpression of ATG5 was confirmed by Western blotting.

### 4.5. qPCR

2 × 10^6^ HL-60-ATG5 cells and mock control cells were collected, and total RNA was isolated, using the Universal RNA Purification Kit (EURx, Gdansk, Poland). The concentration and purity of RNA was evaluated spectrophotometrically using a Nanodrop. Equal amounts of RNA (1 ug) were transcribed into complementary DNA (cDNA) using oligo(dT) primer and Avian Myeloblastosis Virus (AMV) reverse transcriptase (EURx, Gdansk, Poland). qPCR was carried out using gene-specific primers (Table 2), cDNAs, and LightCycler 480 SYBRGreen I Master kit (Roche, Basel, Switzerland) according to the manufacturer’s recommendations using a LightCycler 480 II device (Roche). The results were normalized to two reference genes, actin β (*ACTB*) and hypoxanthine-guanine phosphoribosyltransferase (*HPRT*), and analyzed with the LightCycler^®^ 480 Software.

### 4.6. Western Blot

HL-60 cells were lysed in RIPA buffer supplemented with a protease inhibitor cocktail. Protein concentration was measured using Quick Start Bradford 1× Dye Reagent (Bio-Rad, Hercules, CA, USA). Lysates were sonicated, boiled for 5 min at 95 °C in 5× Laemmli buffer and equal amounts of protein were separated by sodium dodecyl sulphate-polyacrylamide gel electrophoresis (SDS-PAGE) and transferred to the nitrocellulose membrane. Membranes were blocked with 5% milk for 1 h at room temperature (RT) and incubated with primary antibodies, anti-LC3A/B (1:1000 in 1% BSA overnight at 4 °C), anti-ATG5 (1:1000 in 5% BSA overnight at 4 °C), and subsequently with secondary antibodies conjugated with HRP (#7074 at 1:2000, 1 h, RT). Anti-GAPDH or anti-ACTB antibodies conjugated with HRP (1:50,000 in 5% milk, 1 h incubation at RT) were used as loading controls.

### 4.7. NETs Quantification

Differentiated HL-60 cells were seeded into 24-well plates (5 × 10^4^ cells per well) and allowed to settle for 30 min at 37 °C, 5% CO_2_. Subsequently, cells were stimulated with 100 nM PMA or 4 µM CI and incubated for 3 h. Unstimulated cells were used as negative controls. Following incubation, 500 mIU of micrococcal nuclease was added to detach the DNA from the cell surface and the plate was incubated for 20 min at 37 °C, 5% CO_2_. The reaction was then stopped with 5 mM EDTA and the plates were centrifuged (10 min at 415 g). Subsequently, the supernatant was collected, and 100 nM SYTOX green fluorescent dye was added. Extracellular DNA release was measured fluorometrically using FLUOstar Omega plate reader (BMG Labtech, Ortenberg, Germany).

### 4.8. NETs Visualization

To visualize NETs release in vitro, dHL-60 cells were seeded into 48-well plates (2.5 × 10^4^ cells per well) and stimulated with 100 nM PMA or 4 µM CI. After 3 h, 100 nM Sytox Green was added and DNA of compromised cell membranes was visualized under a Leica DMi8 fluorescent microscope (Leica, Wetzlar, Germany) equipped with a 40× and a 10× magnification objectives.

### 4.9. Oxidative Burst Measurement

Differentiated HL-60 cells were incubated with 4 µg/mL dihydrorodamine (DHR)123 for 30 min in 37 °C in the darkness, then washed and seeded into the wells of black 96-well plates (1 × 10^5^ cells per well). Cells were allowed to settle and then stimulated with PMA. Analysis of fluorescence intensity of DHR123 was measured with FLUOstar Omega plate reader (BMG Labtech, Ortenberg, Germany) every 15 min for 2.5 h.

### 4.10. Phagocytosis Assay

Differentiated HL-60 cells (5 × 10^5^) were incubated for 30 min (37 °C, 5% CO_2_) with 25 µg *E. coli* Bio Particles conjugated with fluorescein isothiocyanate (FITC). After incubation, trypan blue solution was added and samples were centrifuged, and subsequently washed twice by 5-min centrifugation at 250 g. The analysis was carried out using BD LSRFortessa flow cytometer and Diva Software (BD Biosciences, San Jose, CA, USA).

### 4.11. Proliferation Assay

Proliferation potential of HL-60 cell lines was measured by flow cytometry and VPD450 staining. HL-60 cells (5 × 10^6^ cells/mL) were suspended in phosphate-buffered saline (PBS) and stained with VPD450 (1 μM) for 15 min at 37 °C, 5% CO_2_. Subsequently, cells were washed with PBS and seeded in 6-well plates and allowed to grow for 5 consecutive days. The flow cytometry analysis of proliferating cells was carried out on the first, third and fifth day of the experiment using BD LSRFortessa flow cytometer and Diva Software.

### 4.12. Phosphatidylserine Externalization

Phosphatidylserine plasma membrane externalization was detected with Annexin V-FITC Apoptosis detection Kit (eBioscience, San Diego, CA, USA). HL-60 cells were seeded in 12-well plates (2.5 × 10^5^ cells per well) and incubated with actinomycin D (1 µg/mL) or H_2_O_2_ (10 µM) for 16 h at 37 °C, 5% CO_2_. After incubation, cells were collected, washed with PBS, resuspended in Binding Buffer and stained with annexin V and PI. The percentage of apoptotic cells was analyzed using BD LSRFortessa flow cytometer and BD FACSDiva Software.

### 4.13. Statistical Analysis

All data were analyzed using GraphPad Prism Software 8 (GraphPad Software, La Jolla, CA, USA). Differences between multiple groups were compared with a Kruskal–Wallis test; for data without normal distribution, or one-way ANOVA for normally distributed data, *p* ≤ 0.05 was considered as significant.

## Figures and Tables

**Table 1:** Sequence of primers used for cloning.

Primer Sequence (5′–3′)
**For *ATG5NotI***: GCGCGGCCGCGCCACCATGACAGA
**Rev *ATG5BamHI***: GCGGATCCTCAATCTGTTGC

Restriction enzymes’ cleavage sites are underlined, For—forward, Rev—reverse.

**Table 2:** Sequences of primers used for qPCR.

Primer Sequences (5′–3′)		
Gene Name	Forward	Reverse
*ATG5*	GGACGAAACAGCTTCTGAAT	GATGGGATTGCAAAATGACA
*ACTB*	AAATCTGGCACCACACCTTC	GGGGTGTTGAAGGTCTCAAA
*HPRT*	GACCAGTCAACAGGGGACAT	AACACTTCGTGGGGTCCTTTTC

*ATG5*—autophagy-related (ATG) 5, *ACTB*—actin β, *HPRT*—hypoxanthine-guanine Phosphoribosyltransferase.
